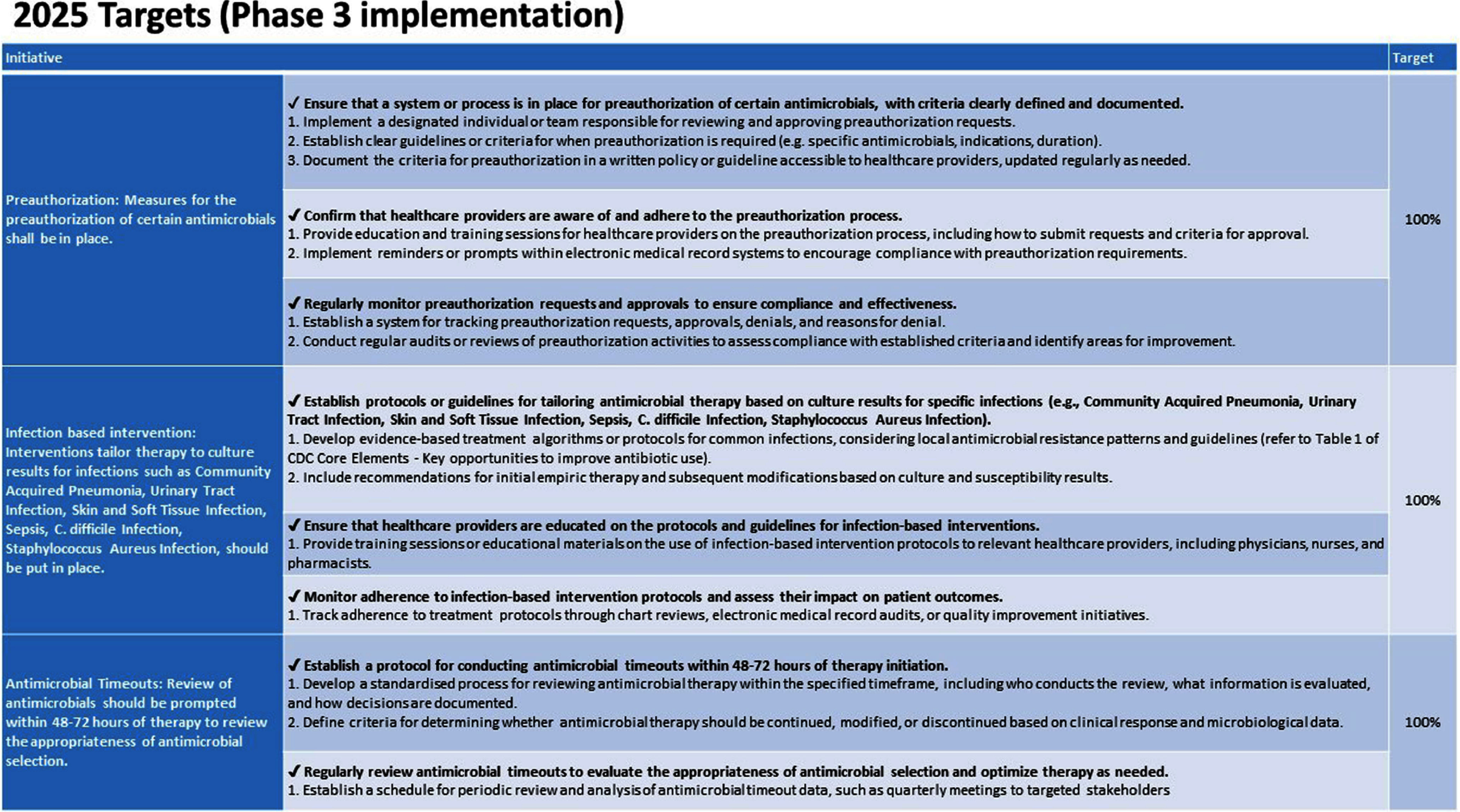# Implementing a Comprehensive Antimicrobial Stewardship Program in a Global Healthcare Organization: A Phased Approach to Sustainable QI

**DOI:** 10.1017/ash.2025.260

**Published:** 2025-09-24

**Authors:** Yiwei Ng, Keith Lim

**Affiliations:** 1 IHH Healthcare; 2 IHH Healthcare

## Abstract

**Background:** Antimicrobial resistance (AMR) is a pressing global public health issue, and the limited development of new antibiotics necessitates robust Antimicrobial Stewardship Programs (ASP). As a global healthcare leader, IHH Healthcare successfully implemented ASP across 80 hospitals in seven countries (Singapore, Malaysia, India, Brunei, Hong Kong, China, and Turkey), aligned with the Centre for Disease Control and Prevention (CDC) Hospital ASP Core Elements, World Health Organization, and national guidelines. **Method:** A three-phase ASP strategy was developed following a crosswalk analysis of ASP practices across the seven countries (See Table 1): Phase 1 (2023): ASP committee establishment, terms of reference, and adoption of evidence-based guidelines. Phase 2 (2024): Guideline compliance audits, antibiogram development, resistance pattern monitoring, post-prescription audits, therapy optimization, and education. Phase 3 (2025): Antimicrobial preauthorization, infection-based interventions, and antimicrobial timeouts within 48–72 hours of initiation. Quarterly ASP meetings facilitated progress tracking and shared learning. Key metrics included guideline adherence, resistance trends, and antimicrobial utilization. **Results:** By 2023, all countries have established ASP committees and adopted guidelines for infections and surgical prophylaxis (see Table 2). In 2024, Phase 2 implementation (see Table 3) showed that: Guideline compliance: Regular audits monitored antimicrobial use for appropriateness, quantity, duration, and type, achieving full compliance across facilities. Education: Comprehensive initiatives included patient education on completing antibiotic regimens and continuous education for healthcare professionals. Post-prescription audits: Standardized protocols ensured systematic audits, with findings and targeted interventions shared with stakeholders. Antibiogram and resistance monitoring: Standardized antibiogram protocols monitored resistance patterns, guiding treatment decisions and policy updates. A framework for tracking key resistance organisms and hospital-acquired infections was also established. Therapy optimization: Policies required prescribers to document antibiotic doses and indications, while IV-to-oral conversion protocols reduced costs and improved outcomes. Metrics like Days of Therapy and Defined Daily Doses measured impact, with dose optimization improving treatment for resistant organisms. **Conclusion:** IHH Healthcare is the first large international group to adopt and implement the U.S. CDC ASP elements across its network of foreign hospitals. By utilizing a phased approach, we have ensured consistent and effective implementation across diverse healthcare settings. To date, all 80 hospitals have successfully completed Phase 2 of the program and are on track to achieve Phase 3 milestones by 2025 (see Table 4). Early outcomes from this initiative underscore the significant value of standardized ASPs in enhancing patient safety, reducing AMR and fostering sustainable quality improvement.

**Figure:**
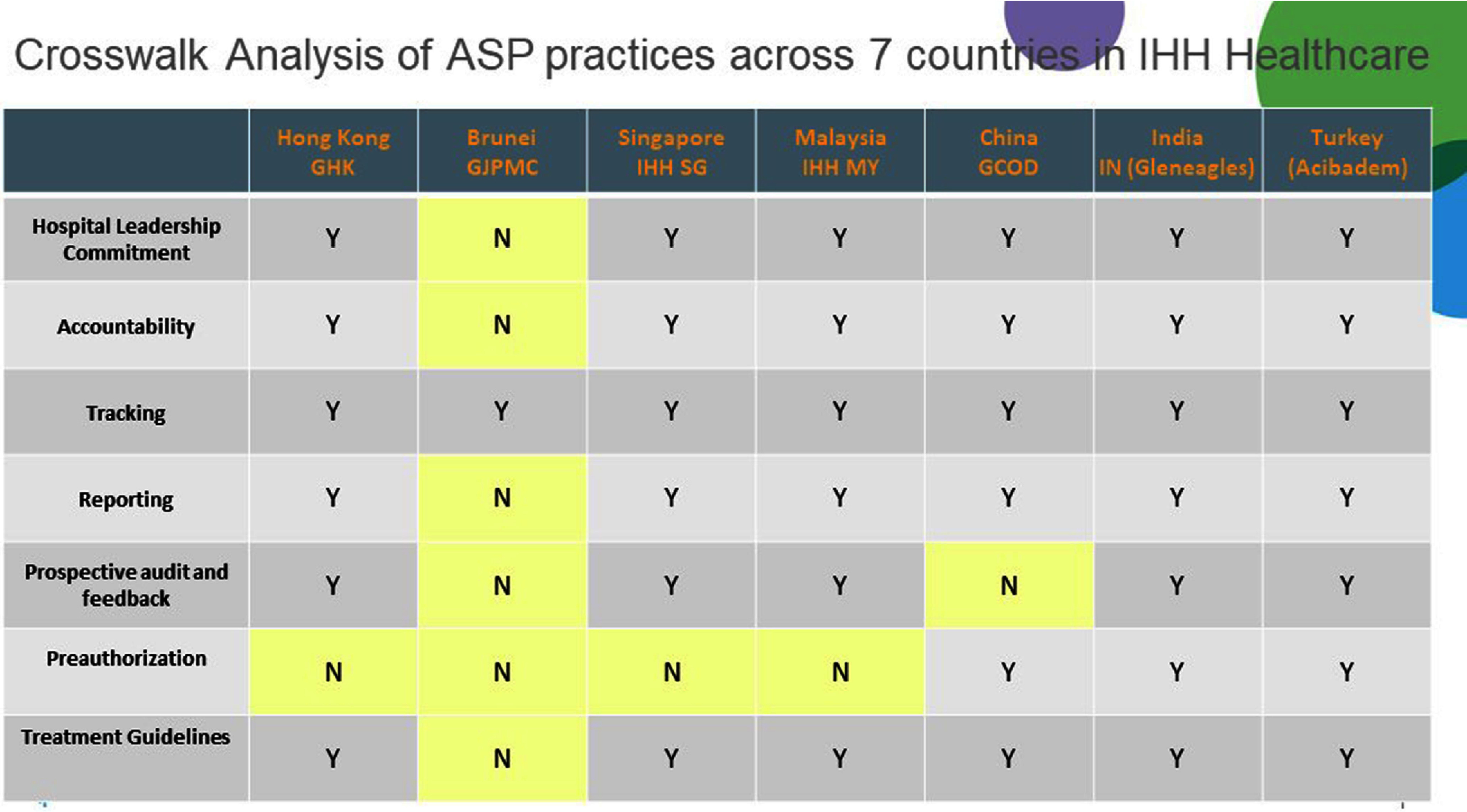

**Figure:**
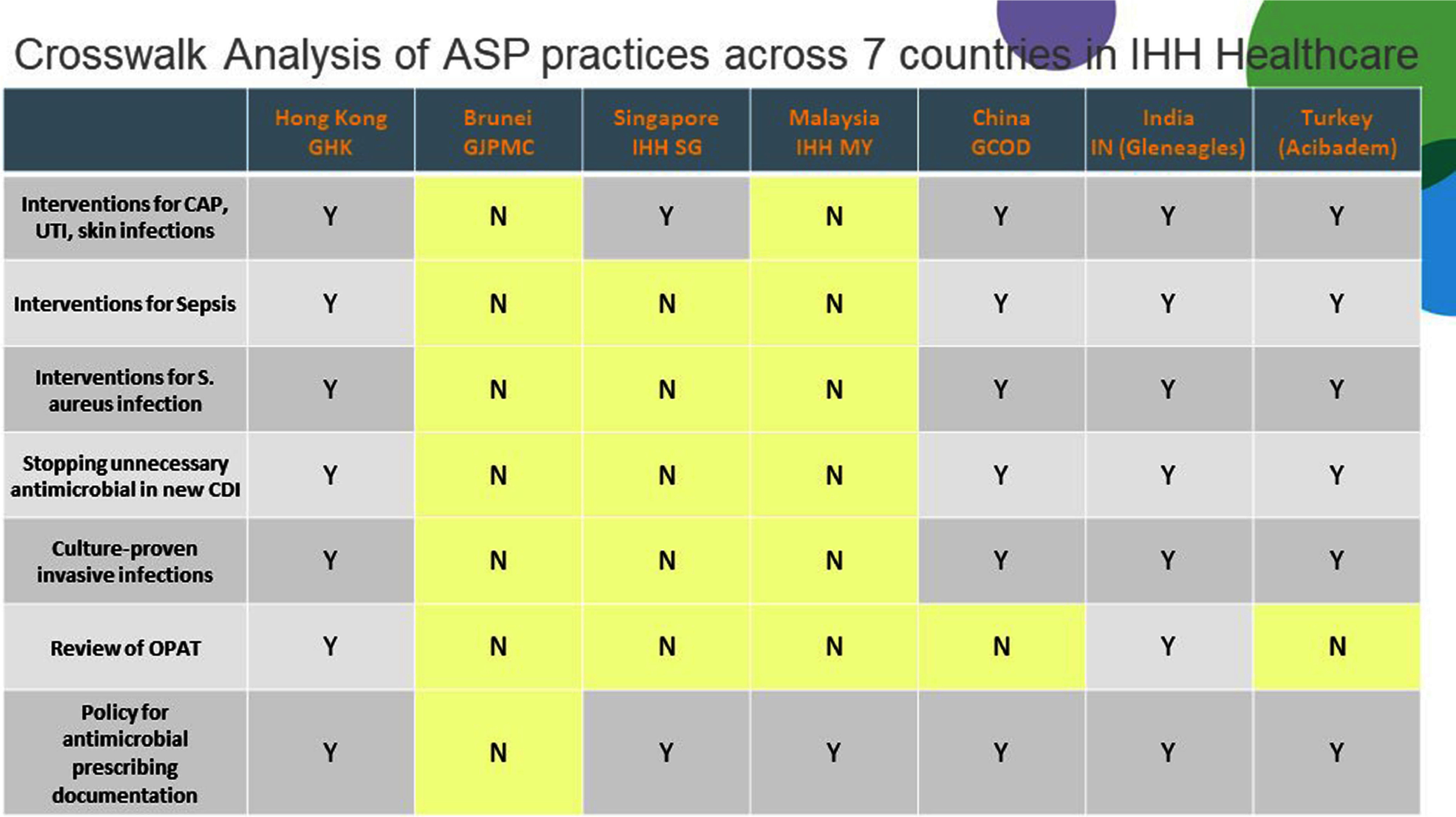

**Figure:**
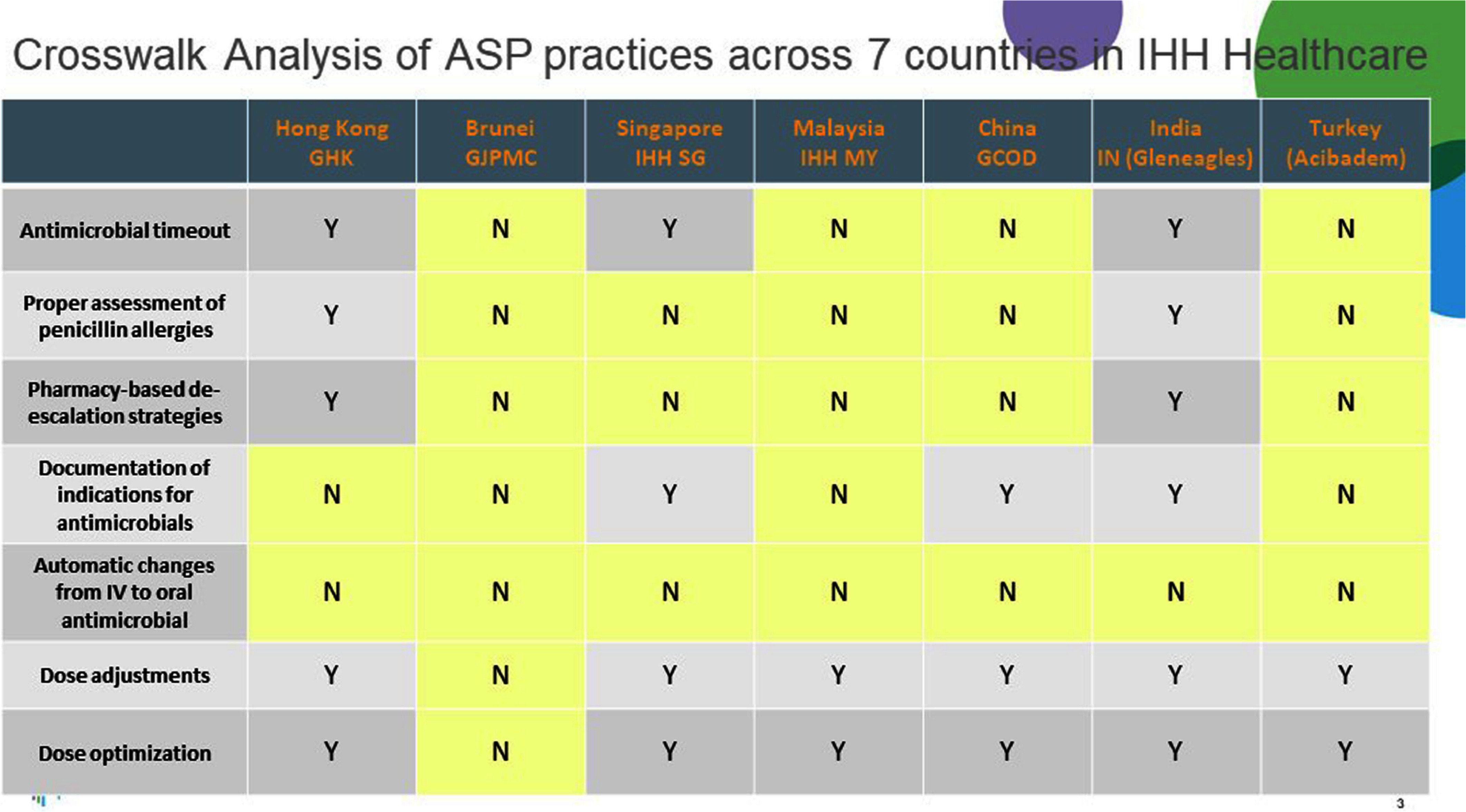

**Figure:**
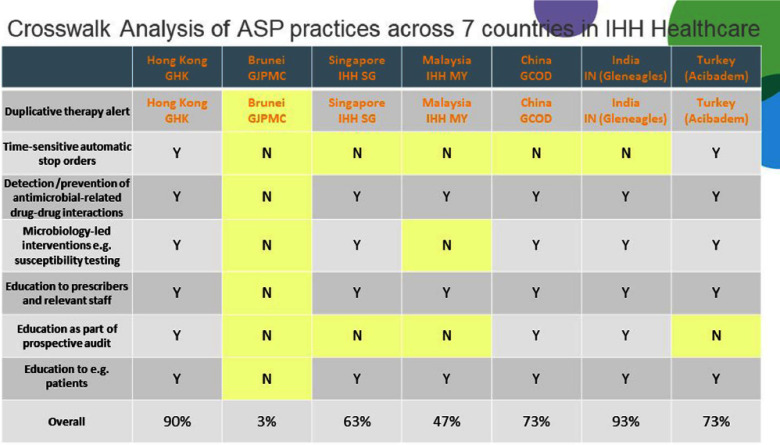

**Figure:**
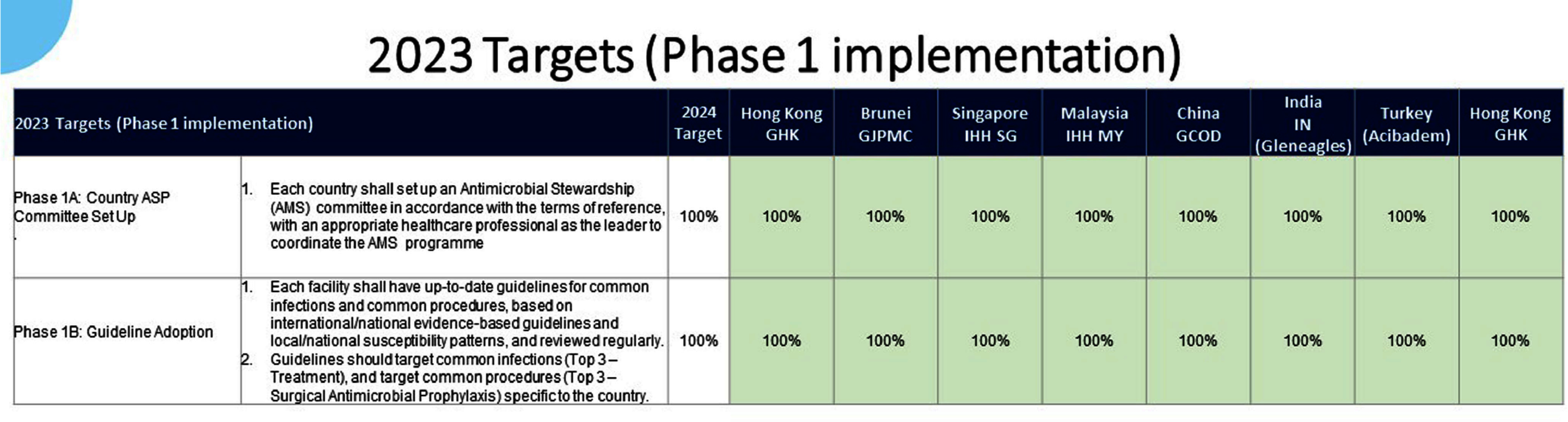

**Figure:**
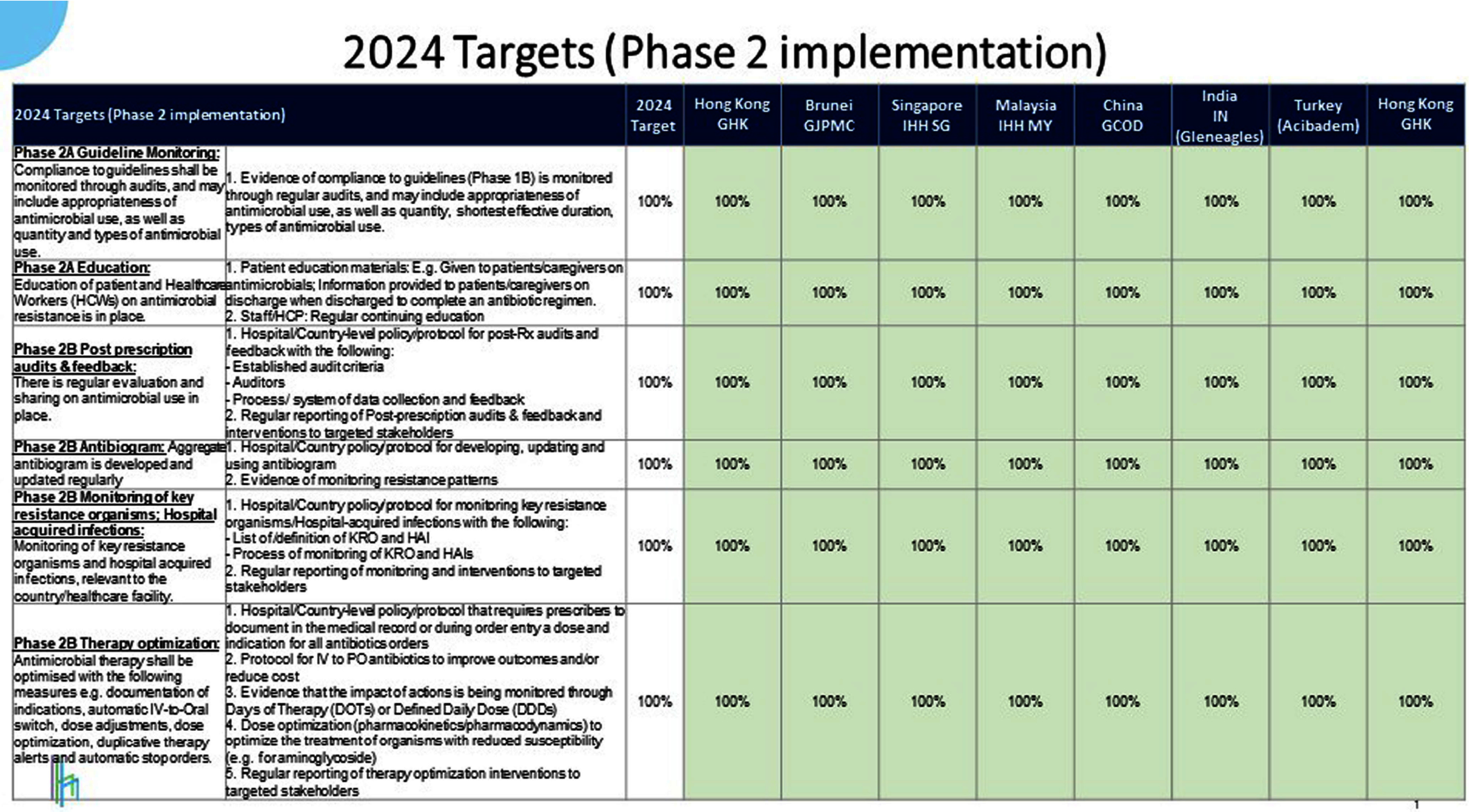

**Figure:**